# Sequential Injection Analysis Method for the Determination of Glutathione in Pharmaceuticals

**DOI:** 10.3390/s24175677

**Published:** 2024-08-31

**Authors:** Maja Biocic, Tomislav Kraljević, Tony G. Spassov, Lea Kukoc-Modun, Spas D. Kolev

**Affiliations:** 1 Department of Analytical Chemistry, Faculty of Chemistry and Technology, University of Split, Rudjera Boskovica 35, 21000 Split, Croatia; maja.biocic@ktf-split.hr; 2 Department of Chemistry, Faculty of Science and Education, University of Mostar, Matice hrvatske bb, 88000 Mostar, Bosnia and Herzegovina; tomislav.kraljevic325@gmail.com; 3 Faculty of Chemistry and Pharmacy, Sofia University “St. Kl. Ohridski”, 1 James Bourchier Blvd., 1164 Sofia, Bulgaria; tspassov@chem.uni-sofia.bg; 4 School of Chemistry, The University of Melbourne, Parkville, VIC 3010, Australia; 5 Department of Chemical Engineering, The University of Melbourne, Parkville, VIC 3010, Australia

**Keywords:** sequential injection analysis, glutathione, neocuproine, pharmaceuticals

## Abstract

A sequential injection analysis method for the determination of glutathione (GSH) in pharmaceuticals has been developed. It is based on the reduction of the Cu(II)-neocuproine complex by GSH and the formation of an orange-yellow colored Cu(I)-neocuproine complex with maximum absorbance at 458 nm. Under optimal conditions the method is characterized by a linear calibration range of 6.0 × 10^−7^–8.0 × 10^−5^ mol L^−1^ (*A_max_* = 3270 *C_GSH_* − 0.0010; *R*^2^ = 0.9983), limit of detection of 2.0 × 10^−7^ mol L^−1^, limit of quantification of 6.7 × 10^−7^ mol L^−1^, repeatability (expressed as relative standard deviation) of 3.8%, and sampling rate of 60 h^−1^. The newly developed method has been successfully applied to the determination of GSH in pharmaceutical samples with no statistically significant difference between the results obtained and those produced by the standard Pharmacopoeia method.

## 1. Introduction

Glutathione (GSH) is a protein synthesized in the liver that plays an essential role in intercellular metabolism and the organism’s immune system. It protects cells from oxidative stress by reducing reactive oxygen species [1,2]. GSH is a tripeptide consisting of three amino acids, i.e., glycine, cysteine, and glutamic acid. The sulfhydryl group (−SH) of cysteine in GSH plays a crucial role in conjugation and reduction reactions. GSH comes in two forms: reduced (GSH) and oxidized (GSSG), and the ratio of these two forms can determine the oxidation state of the cell [3]. It is commonly used in the treatment of Parkinson’s disease and as an antidote to heavy metal poisoning [4]. The interest in glutathione as an analyte has increased since the end of the 20th century when scientific interest in the pronounced antioxidant effect of glutathione in the body began.

A number of analytical methods have been reported in the literature for the determination of GSH, most of which are designed for analyzing GSH in biological fluids and pharmaceutical formulations. These methods are often based on the use of spectrophotometry [5,6,7,8,9], fluorimetry [10,11,12,13,14], high-performance liquid chromatography (HPLC) [15,16,17,18,19,20], atomic absorption spectroscopy (AAS) [21], voltammetry [22], and kinetic methods with spectrophotometric [23,24] or potentiometric [25] detection. Some of the developed methods often involve the determination of both the reduced and oxidized forms of GSH [10,15]. However, not all of the methods mentioned above are suitable for direct routine analysis of GSH. Some of the methods are time-consuming and require laborious and/or time-consuming sample pretreatment or processing which results in low sampling rates [10,11,15,16]. 

Continuous-flow manufacturing, based on dynamic flow technology, offers more flexibility than traditional batch processes, making it an attractive alternative to these batch processes in the pharmaceutical industry [26]. Continuous manufacturing requires continuous monitoring to ensure production conditions are not compromised over time [26]. Continuous monitoring can be carried out by in-line or on-line mode. In in-line mode the analytical instrument is an integral part of the manufacturing setup and monitoring usually involves continuous flow of process solution through a flow-through detector. In on-line mode, a sample of the process solution is periodically analyzed by the automated analytical instrument. Flow analysis techniques are particularly suitable for on-line process monitoring and analysis [27].

Flow injection analysis (FIA), as the first generation of flow analysis techniques, has revolutionized chemical analysis by reducing drastically the volumes of samples and reagents required and by conducting fast sample processing online [28]. FIA methods utilizing different detectors (e.g., spectrophotometric [29,30,31], chemiluminescence [32,33,34,35,36,37], amperometric [38,39,40,41], voltammetric [42], and potentiometric [43]), have been reported in the literature. These methods have become versatile and cost-effective tools for pharmaceutical quality control in recent decades due to their simplicity and high throughput capacity [44]. In addition, FIA promotes greener analytical chemistry by enabling the development of environmentally friendly and safe analytical methods in terms of low waste generation and minimal human participation in the analytical process [45].

Sequential injection analysis (SIA) is the second generation of flow analysis techniques that was developed in 1990 by Ruzicka and Marshall [28] as an environmentally friendlier alternative to FIA because SIA methods require even smaller reagent and sample volumes than FIA thus further reducing chemical waste and cost of analysis. SIA methods also simplify the conduction of complex analytical reactions in on-line mode.

Despite the advantage of SIA compared to FIA only two SIA methods for the determination of GSH have been reported so far [46,47]. The first method involves enzymatic reactions for detecting oxidized and reduced glutathione in human blood spectrophotometrically at 412 nm using 2-nitro-5-thiobenzoic acid [46]. The second method involves the reaction of GSH with ethyl propiolate to form a thioacryl derivative that can be detected spectrophotometrically in the ultraviolet range [47]. These two methods use toxic (i.e., 2-nitro-5-thiobenzoic acid) or expensive (i.e., enzymes) reagents and the detection in one of them is done in the UV range where significant interferences could be encountered. Therefore, there is a need for a SIA method based on a one-step analytical reaction, utilizing inexpensive and non-toxic reagents, with detection in the visible range.

The purpose of this study is to develop such a SIA method for the determination of GSH in pharmaceuticals using spectrophotometric detection in the visible range. The newly developed method is based on a one-step redox analytical reaction in which GSH reduces the copper(II)-neocuproine complex to an orange-yellow colored copper(I)-neocuproine complex (Equation (1)) with an absorption maximum at 458 nm.

2GSH + 2[Cu(Nc)_2_]^2+^ ⇄ GSSG + 2[Cu(Nc)_2_]^+^ + 2H^+^ (1)

## 2. Materials and Methods

### 2.1. Solution Preparation

Analytical-grade reagents were used in this study without any further purification. Solutions were prepared in deionized water (Milli-Q, Millipore, Bay City, MI, USA, 18 MΩ).

The GSH stock solution with a concentration of 1.0 × 10^−2^ mol L^−1^ was prepared by dissolving 0.3073 g GSH (Sigma-Aldrich, Saint Louis, MO, USA) in Britton-Robinson buffer solution (pH = 2) and then the solution was diluted to 100 mL. The stock solution was stored at 4 °C in a dark bottle where it remained stable for at least one month. Working standards were prepared daily by diluting the stock solution with Britton-Robinson buffer solution (pH = 3). The water solubility of neocuproine (2,9-dimethyl-1,10-phenantroline) was improved by reacting it with Cu(II) to form the water-soluble copper(II)-neocuproine, (Cu(Nc)_2_^2+^) complex [48]. The Cu(Nc)_2_^2+^ reagent was prepared by dissolving 25.0 mg of CuSO_4_ × 5H_2_O (Kemika, Zagreb, Croatia) and 50.0 mg of neocuproine hydrate (Sigma-Aldrich, Saint Louis, MO, USA) in 100 mL of Britton-Robinson buffer solution (pH = 3) [24]. The Cu(Nc)_2_^2+^ reagent was stable at 4 °C for at least a month. The concentrations of copper(II) and neocuproine in the reagent solution were 1.0 × 10^−4^ mol L^−1^ and 2.4 × 10^−4^ mol L^−1^, respectively, which produced a molar ratio of *n*(Cu^2+^):*n*(Nc) = 1:2.4 as recommended for the determination of GSH with this reagent in a previous kinetic batch study [24].

The newly developed method was validated with the determination of GSH in commercial pharmaceutical capsules, containing 50 mg GSH per capsule. In these analyses, five capsules were weighed and mixed in powder form. An amount of powder corresponding to 50 mg GSH was dissolved in 500 mL deionized water and after appropriate dilution, the solution was analyzed without delay to avoid possible GSH oxidation. The concentration of GSH in the same solution was determined by back-titration using standard solutions of iodine (Kemika, Zagreb, Croatia) and thiosulfate (Kemika, Zagreb, Croatia) in accordance with the British Pharmacopeia procedure [49].

### 2.2. Apparatus and Analytical Procedure

**Figure 1:**
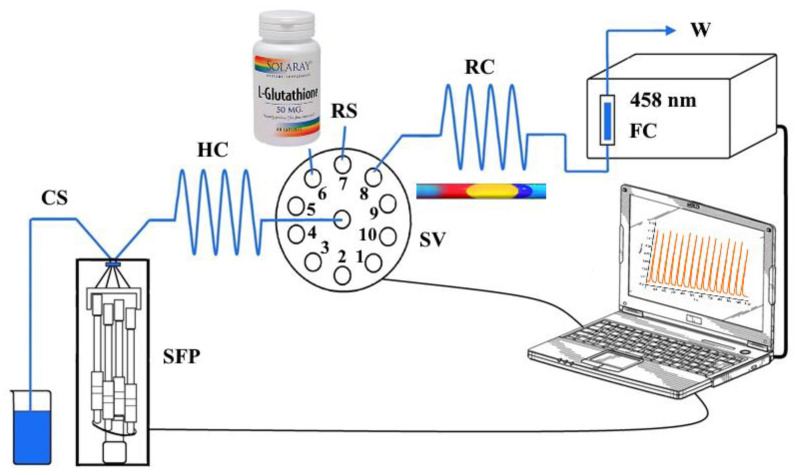
Schematic diagram of the SIA system: SFP—syringe-free pump (M50), SV—10-port selection valve, HC—holding coil, RC—reaction coil, CS—carrier stream (deionized H_2_O), RS—reagent stream, FC—flow cell, W—waste.

The home-made SIA system (Figure 1) used to determine GSH consisted of a bi-directional stepper-motor syringe-free pump (Cheminert^®^ M50, VICI Valco Instruments, Houston, TX, USA) which was connected to a programmable micro-stepping driver (Micro Lynx 4, Intelligent Motion Systems, Phoenix, AZ, USA), and a 10-port selection valve (C25-3180EMH, VICI Valco Instruments, Houston, TX, USA) equipped with a multi-position actuator control module (EMHCA-CE, VICI Valco Instruments, Houston, TX, USA). These system components were controlled by M6-LHS software (VICI Valco Instruments, Houston, TX, USA). All system tubing, including that in the holding coil (HC, 120 cm) and in the reaction coil (RC, 30 cm) was made of PTFE and had an internal diameter of 0.8 mm. The absorbance measurements at 458 nm were conducted by a double beam UV-Vis spectrophotometer (UV–1601, Shimadzu, Tokyo, Japan) equipped with a flow cell (10 mm optical path, internal volume of 80 µL, Hellma, USA). The spectrophotometer was connected to a personal computer running UVProbe software, Version 2.42 (Shimadzu, Tokyo, Japan). The absorbance of the reduced orange-yellow [Cu(Nc)_2_]^+^ complex (Equation (1)) was measured with a frequency of 1 Hz at its maximum absorbance wavelength of 458 nm.

The measurements were carried out by aspirating defined volumes of reagent and sample solutions into the holding coil where they formed adjacent zones. These zones were propelled towards the flow-through cuvette of the spectrophotometer via the reaction coil (Figure 1). The SIA absorbance peak value was used as the analytical signal. The optimal solution volumes and flow rates are listed in Table 1. 

## 3. Results and Discussion

The optimal composition of the reagent solution (i.e., pH 3 and concentrations of Cu(II) and neocuproine of 1.0 × 10^−4^ mol L^−1^ and 2.4 × 10^−4^ mol L^−1^, respectively) was previously determined in a kinetic batch study [24]. GSH is a thiol compound and therefore it is more stable in acidic media, and its reaction with copper(II)-neocuproine complex is not affected by temperature variations in a wide range (i.e., 5–60 °C) [24]. Therefore, there was no need for thermostating the SIA system which operated at room temperature of 25 ± 1 °C.

### 3.1. Optimization of the SIA System Parameters

**Figure 2:**
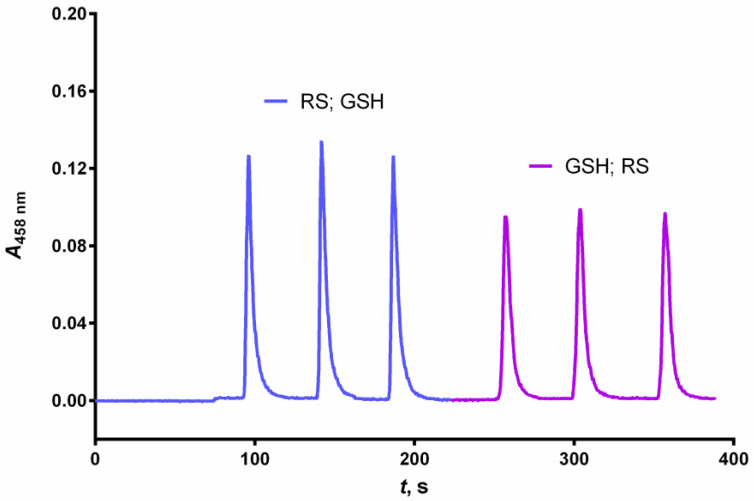
Siagram of the aspiration sequence of analyte (GSH) and reagent (RS) solutions. Experimental conditions: *c*(GSH) = 4 × 10^−5^ mol L^−1^, *c*(Cu^2+^) = 1.0 × 10^−4^ mol L^−1^, *c*(Nc) = 2.4 × 10^−4^ mol L^−1^, pH = 3.0, temperature = 25 °C, carrier flow rate = 5000 μL min^−1^, reagent volume = 200 μL, sample volume = 200 μL, volume of the holding coil = 1000 µL, length of the reaction coil = 65 cm.

Two different aspiration sequences of the GSH standard (200 μL) and reagent (200 μL) solution were compared during the optimization process. The first sequence involved aspirating the GSH standard solution followed by aspiration of the reagent solution. The second sequence involved aspirating the reagent solution followed by aspirating the GSH standard solution. The second sequence produced a 25% higher analytical signal (Figure 2) due to the shorter travel distance of the GSH standard resulting in its lower dilution and hence higher absorbance peaks [50]. This order of aspiration was maintained for all subsequent measurements.

**Figure 3:**
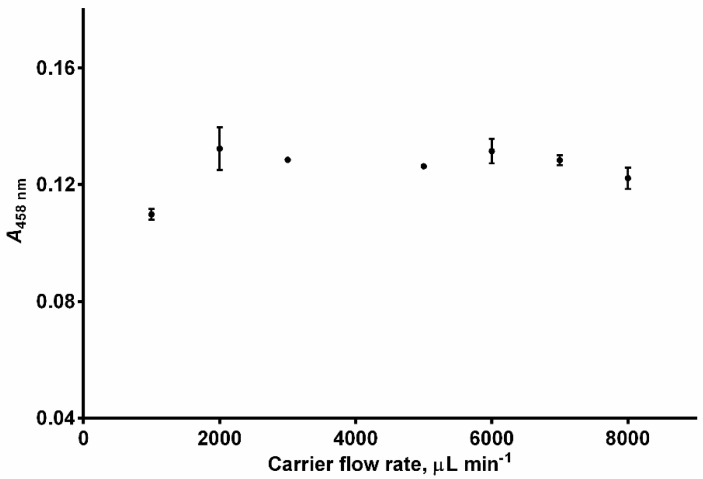
Effect of the carrier flow rate on peak absorbance (*A*_458 nm_). Experimental conditions: *c*(GSH) = 4 × 10^−5^ mol L^−1^, *c*(Cu^2+^) = 1 × 10^−4^ mol L^−1^, *c*(Nc) = 2.4 × 10^−4^ mol L^−1^, pH = 3.0, temperature = 25 °C, reagent volume = 200 μL, sample volume = 200 μL, volume of the holding coil = 1000 μL, length of the reaction coil = 65 cm. Error bars = ±standard deviation (SD) (n = 3).

The flow rate effect on the analytical signal was examined in the range from 1000 μL min^−1^ to 8000 μL min^−1^. The analytical signal increased when the flow rate was increased from 1000 to 2000 μL min^−1^ and then levelled off (Figure 3). Due to the better repeatability at 3000 μL min^−1^ compared to that at 2000 μL min^−1^, the former flow rate was selected for the subsequent experiments.

**Figure 4:**
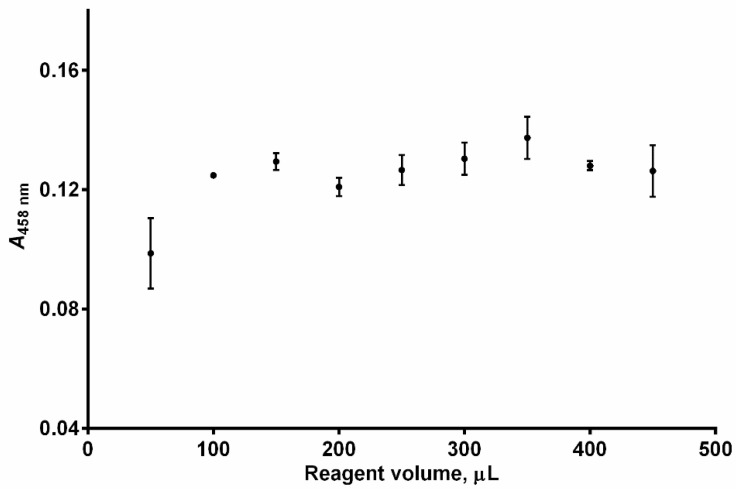
Influence of the reagent volume on peak absorbance (*A*_458 nm_). Experimental conditions: *c*(GSH) = 4 × 10^−5^ mol L^−1^, *c*(Cu^2+^) = 1 × 10^−4^ mol L^−1^, *c*(Nc) = 2.4 × 10^−4^ mol L^−1^, pH = 3.0, temperature = 25 °C, carrier flow rate = 3000 mL min^−1^, sample volume = 200 μL, volume of the holding coil = 1000 μL, length of the reaction coil = 65 cm. Error bars = ±SD (n = 3).

The reagent volume was varied from 50 μL to 450 μL (Figure 4). The analytical signal increased with the reagent volume up to 150 μL and then levelled off. Therefore, this value was selected for all subsequent experiments.

**Figure 5:**
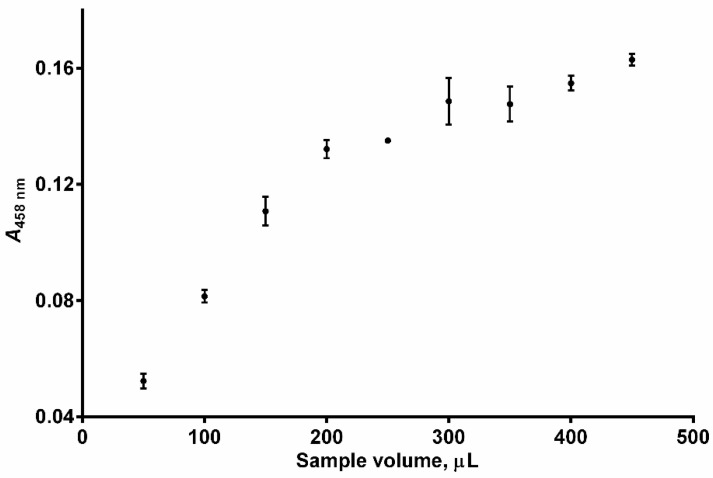
Effect of the sample volume on peak absorbance (*A*_458 nm_). Experimental conditions: *c*(GSH) = 4 × 10^−5^ mol L^−1^, *c*(Cu^2+^) = 1 × 10^−4^ mol L^−1^, *c*(Nc) = 2.4 × 10^−4^ mol L^−1^, pH = 3.0, temperature = 25 °C, carrier flow rate = 3000 mL min^−1^, reagent volume = 150 μL, volume of the holding coil = 1000 μL, length of the reaction coil = 65 cm. Error bars = ±SD (n = 3).

As expected, the analytical signal increased with the sample volume in the studied range (i.e., 50–450 μL) (Figure 5). However, for volumes higher than 200 μL this increase became less pronounced and therefore it was decided that a reasonable compromise between signal height and sample consumption was 200 μL.

The analytical signals corresponding to two different holding coil (HC, Figure 1) volumes (i.e., 500 and 1000 μL), each being significantly larger than the combined volume of the reagent and sample, were found to be statistically insignificantly different at the 95% confidence level. Therefore, a 500 μL holding coil was used in the subsequent experiments.

**Figure 6:**
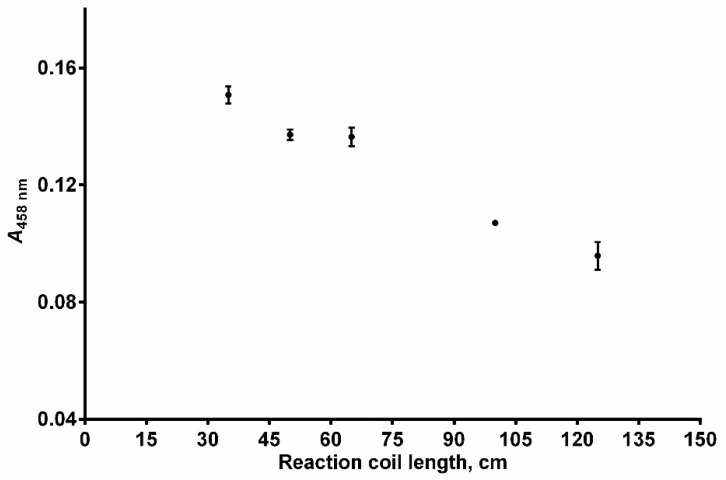
Effect of the reaction coil length on peak absorbance (*A*_458 nm_). Experimental conditions: *c*(GSH) = 4 × 10^−5^ mol L^−1^, *c*(Cu^2+^) = 1 × 10^−4^ mol L^−1^, *c*(Nc) = 2.4 × 10^−4^ mol L^−1^, pH = 3.0, temperature = 25 °C, carrier flow rate = 3000 mL min^−1^, reagent volume = 150 μL, sample volume = 200 μL, volume of the holding coil = 500 μL. Error bars = ±SD (n = 3).

The influence of the reaction coil length (RC, Figure 1) on the analytical signal was investigated in the range of 35–125 cm. Though the highest analytical signal was observed at 35 cm length, better reproducibility with an insignificant decrease in the analytical signal was obtained at 50 cm length (Figure 6). Hence, a reaction coil of 50 cm in length was selected for the final SIA system configuration.

### 3.2. Analytical Figures of Merit

**Figure 7:**
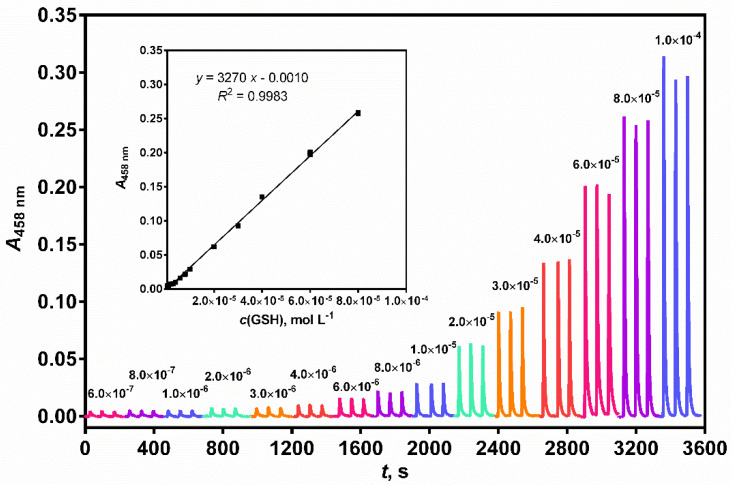
Siagrams of the spectrophotometric determination of GSH in the concentration range of 6.0 × 10^−7^ mol L^−1^ to 1.0 × 10^−4^ mol L^−1^. Inset: calibration curve for the SIA determination of GSH in the range 6.0 × 10^−7^ mol L^−1^ to 8.0 × 10^−5^ mol L^−1^, obtained under optimal conditions (Table 2).

The newly developed method was calibrated in the range 6.0 × 10^−7^–1.0 × 10^−4^ mol L^−1^ using the optimized parameters of the SIA method and 15 GSH standards (Table 2). Significant deviation from linearity was observed for the 1.0 × 10^−4^ mol L^−1^ standard and therefore, a linear calibration curve (*A_458 nm_* = 3.27 × 10^3^ *C_GSH_* − 1 × 10^−3^, *R*^2^ = 0.9983) was obtained in the concentration range of 6.0 × 10^−7^–8.0 × 10^−5^ mol L^−1^ (Figure 7). The limit of detection (LOD) and limit of quantification (LOQ) based on 3 times and 9 times the standard deviation of the blank were calculated as 2.0 × 10^−7^ mol L^−1^ and 6.7 × 10^−7^ mol L^−1^, respectively. The repeatability, expressed as the relative standard deviation (RSD) of 30 replicate measurements of a 4 × 10^−5^ mol L^−1^ GSH standard, was calculated as 3.80%. The sampling rate was found to be 60 h^−1^.

The LOD of the newly developed method is approximately 6 times higher than that of the enzymatic method reported by Araujo et al. [46] and comparable to that of Zacharis et al. [47] (Table 3).

However, the former method, which uses an enzymatic reagent, is more expensive, has a significantly narrower working range and its sampling rate is approximately 5 times lower. The newly developed method offers a much wider linear dynamic range of over two orders of magnitude (Table 3). An advantage of the newly developed method compared to that reported by Zacharis et al. [47] is that it operates in the visible range thus avoiding possible interferences by compounds absorbing in the UV range. Such compounds are a number of the excipients used in commercial pharmaceutical formulations (Table 4) (e.g., glucose [51], fructose [52], and sodium citrate [53]).

### 3.3. Effect of Interferences

**Figure 8:**
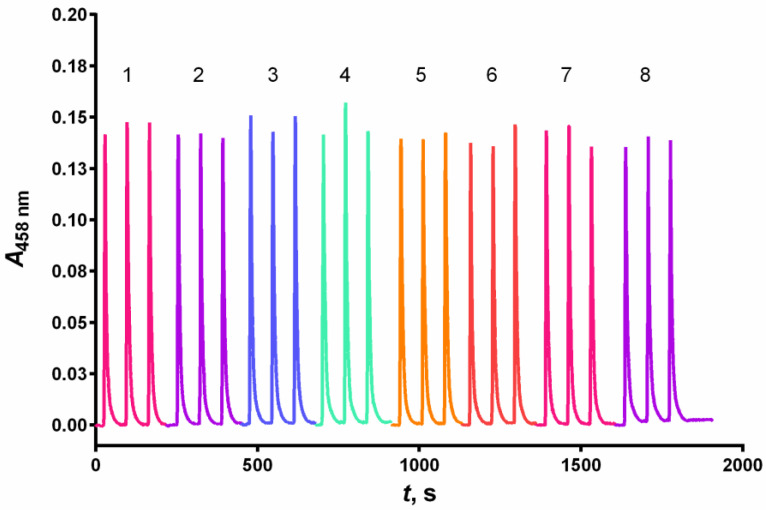
Siagrams of the spectrophotometric determination of 4 × 10^−5^ mol L^−1^ GSH (1) and 4 × 10^−5^ mol L^−1^ GSH in the presence of 2 × 10^−2^ mol L^−1^ (500 times molar excess) glucose (2), fructose (3), lactose (4), KNO_3_ (5), Na_2_SO_4_ (6), boric acid (7), or 4 × 10^−4^ mol L^−1^ (10 times molar excess) sodium citrate, obtained under optimal conditions (Table 2).

Common excipients found in commercial pharmaceutical formulations, including (KNO_3_ and Na_2_SO_4_) and reducing sugars (glucose, fructose, lactose) did not produce an error of more than 5% in the determination of 4.0 × 10^−5^ mol L^−1^ GSH in molar excess of up to 500 (Table 4, Figure 8).

### 3.4. Method Validation

Commercial GSH capsules, which according to the manufacturer contained 50 mg GSH per capsule, were analyzed in triplicate by the newly developed and the reference method [49] and no statistically significant difference at the 95% confidence level was found between the results obtained by both methods (i.e., 50.3 ± 0.9 mg and 52.0 ± 0.7). 

In addition, the recovery values in the range of 96–102% in a recovery study (Table 5), involving the same commercial GSH capsules, further confirmed the validity of the newly developed SIA method.

## 4. Conclusions

The newly developed SIA method for GSH determination, based on a one-step redox reaction and spectrophotometric detection in the visible range is characterized by a wide linear range (6.0 × 10^−7^–8.0 × 10^−5^ mol L^−1^), low LOD of 2.0 × 10^−7^ mol L^−1^, and high sampling rate of 60 h^−1^. It was validated with samples of commercial GSH capsules. Based on the abovementioned characteristics, it can be concluded that this method is an attractive alternative to published SIA methods in terms of costs and selectivity regarding common excipients in commercial pharmaceutical formulations.

## Figures and Tables

**Table 1:** Optimized SIA procedure.

Step	Valve Position	Flow Rate, µL min^−1^	Volume, µL	Description
1	7	3000	150	Aspiration of reagent solution to the holding coil
2	6	3000	200	Aspiration of sample solution to the holding coil
3	8	3000	3000	Propelling of the reaction mixture to the flow cell

**Table 2:** Initial values, studied ranges, and optimal values of the main design and operational parameters of the SIA method (RS-reagent solution, GSH–analyte solution).

Studied Parameters	Initial Value	Studied Range	Optimal Value
Aspiration sequence	RS, GSH	RS, GSH; GSH, RS	RS, GSH
Carrier flow rate, µL min^−1^	5000	1000–8000	3000
Reagent volume, µL	200	50–450	150
Sample volume, µL	200	50–450	200
Holding coil length, μL	1000	500; 1000	500
Reaction coil length, cm	65	35–125	50

**Table 3:** The comparison of the analytical parameters of the presented SIA method for the determination of GSH with the previously developed SIA methods using spectrophotometric detection.

*l*_detection_, nm	Linear Range, mol L^−1^	LOD, mol L^−1^	RSD, %	Sampling Rate, h^−1^	Sample	Ref.
412	1.0 × 10^−7^–3.0 × 10^−6^	3.1 × 10^−8^	<5.00	13	blood	[46]
285	4.9 × 10^−7^–2.3 × 10^−4^	1.6 × 10^−7^	3.00	100	pharmaceuticals	[47]
458	6.0 × 10^−7^–8.0 × 10^−5^	2.0 × 10^−7^	3.80	60	pharmaceuticals	present

LOD—Limit of Detection; RSD—Relative Standard Deviation.

**Table 4:** The effect of excipients commonly used in commercial pharmaceutical formulations in solutions containing 4 × 10^−5^ mol L^−1^ GSH (Peak absorbance; SD (n = 3); 0.145; 0.003).

Excipient	Molar Ratio of GSH to Excipient	Peak Absorbance; SD (n = 3)	Relative Error, %
Glucose	1:500	0.141; 0.001	−2.9
Fructose	1:500	0.148; 0.004	1.8
Lactose	1:500	0.147; 0.009	1.3
KNO_3_	1:500	0.140; 0.002	−3.5
Na_2_SO_4_	1:500	0.140; 0.006	−3.5
Boric acid	1:500	0.142; 0.005	−2.7
Sodium citrate	1:10	0.138; 0.003	−4.9

**Table 5:** Evaluation of the accuracy of the SIA method for the determination of GSH.

Sample	Added, mg	Found ^(b)^, mg	Recovery, %
L-glutathione ^(a)^	0	50.3 ± 0.9	–
	50	96.5 ± 1.1	96.5
	100	149.7 ± 0.5	99.8
	150	195.8 ± 1.3	97.9
	200	254.0 ± 1.5	101.6

^(a)^ Capsules containing GSH 50 mg and excipients. ^(b)^ Average ± standard deviation (SD) of three determinations per sample.

## Data Availability

Data will be provided upon request.
